# Associating the biomarkers of ocular blood flow with lamina cribrosa parameters in normotensive glaucoma suspects. Comparison to glaucoma patients and healthy controls

**DOI:** 10.1371/journal.pone.0248851

**Published:** 2021-03-23

**Authors:** Patrycja Krzyżanowska-Berkowska, Karolina Czajor, D. Robert Iskander

**Affiliations:** 1 Department of Ophthalmology, Wroclaw Medical University, Wroclaw, Poland; 2 Department of Biomedical Engineering, Faculty of Fundamental Problems of Technology, Wroclaw University of Science and Technology, Wroclaw, Poland; Icahn School of Medicine at Mount Sinai, UNITED STATES

## Abstract

**Purpose:**

To evaluate association between ocular blood flow biomarkers and lamina cribrosa parameters in normotensive glaucoma suspects compared to glaucoma patients and healthy controls.

**Methods:**

A total of 211 subjects (72 normotensive glaucoma suspects, 70 with primary open-angle glaucoma and 69 controls) were included. Ocular blood flow biomarkers in ophthalmic artery, central retinal artery, as well as in nasal and temporal short posterior ciliary arteries were measured using colour Doppler imaging. Lamina cribrosa position was assessed by measuring its depth, deflection depth, lamina cribrosa shape index and its horizontal equivalent (LCSI_H_) on B-scan images obtained using optical coherence tomography.

**Results:**

Ocular blood flow biomarkers in glaucoma patients were statistically significantly reduced when compared to healthy controls in peak systolic velocity (PSV) (P = 0.001 in ophthalmic artery and P<0.001 in central retinal artery) and mean flow velocity (V_m_) (P = 0.008 in ophthalmic artery and P = 0.008 in central retinal artery), but not statistically significantly different to that of glaucoma suspects except for PSV in central retinal artery (P = 0.011). Statistically significant correlations corrected for age, central corneal thickness and intraocular pressure were found in glaucoma patients between LCSI_H_ and end diastolic velocity of central retinal artery (P = 0.011), and of nasal short posterior ciliary artery (P = 0.028), and between LCSI_H_ and V_m_ of central retinal artery (P = 0.011) and of nasal short posterior ciliary artery (P = 0.007). No significant correlations were observed between these parameters in glaucoma suspects and healthy controls.

**Conclusions:**

Impaired ocular blood flow associated with the deformation of lamina cribrosa was found in glaucoma patients, whereas glaucoma suspects had similar lamina cribrosa shape to glaucoma patients but that deformation was not associated with ocular blood flow biomarkers.

## Introduction

Glaucoma suspects are the individuals with disease-related risk factors that indicate an increased likelihood of developing primary open-angle glaucoma (POAG) [1]. The major risk factor for developing glaucoma and lamina cribrosa (LC) deformation in the course of disease is elevated intraocular pressure (IOP). Nevertheless, in a large proportion of patients development of glaucoma occurs at IOP within normal limits and the role of vascular risk factors in the pathogenesis of glaucoma is emphasized [2–4]. Moreover, in relation to glaucoma progression evidence for decreased optic nerve blood flow has been reported [5–7]. Because vascular insufficiency of the optic nerve head (ONH) is considered to be an important factor in pathogenesis of glaucoma, it seems reasonable to assess the blood supply of the ONH in glaucoma suspects. However, studies on ocular blood flow (OBF) using colour Doppler imaging (CDI) in normotensive glaucoma suspects are scarce [8–10]. We have already reported the results of OBF velocities in patients with glaucomatous optic disc appearance, noting that the observed similarity of healthy controls and glaucoma suspects and dissimilarity between suspects and POAG groups in terms of pulsatility index/resistive index relationship is remarkable [11]. The development of enhanced depth imaging technique using spectral-domain optical coherence tomography allowed for visualization of the LC and choroid *in vivo*. Consequently, this technique has been used to evaluate these structures in both normal and glaucomatous subjects [12–14]. Additionally, the LC deformation has been recognized as a key phenomenon underlying the pathogenesis of glaucoma [15, 16], but the presence of LC deformation in glaucoma suspects is still under investigation. We recently demonstrated that the deformation of LC in glaucoma suspects is similar to that of glaucoma patients and occurs mainly in the central part of the anterior surface [17]. As noted by Embleton et al. [18], it is likely that a decrease in the LC elasticity affects the ocular blood flow in the supplying ciliary vessels of glaucoma patients. Such a sequence of events has not been investigated in glaucoma suspects. To the best of our knowledge, the relationship between OBF biomarkers and lamina cribrosa position in glaucoma suspects in comparison to those of glaucoma patients and healthy controls has not been established. Hence, continuing our research in normotensive patients with suspected glaucoma, we formulate the hypothesis that the association of OBF biomarkers with morphological features in the region of ONH in the group of normotensive glaucoma suspects is similar to that of POAG patients.

## Materials and methods

### Study subjects

This study included normotensive glaucoma suspects (NGS), patients with POAG and healthy controls (HC). The study was approved by the Bioethical Committee of the Wroclaw Medical University (KB–332/2015) and adhered to the tenets of the Declaration of Helsinki. Informed written consent to participate was obtained from all subjects.

NGS and POAG subjects included into the study, were the patients who reported for regular visits to the Glaucoma Clinic and had complete documentation of optic disc imaging, IOP and visual field testing over three to four years with follow-ups of about 12 months. The results from several visits were considered for assigning a patient to the NGS group. All patients were interviewed for their medical and ophthalmic history. Then, patients underwent comprehensive ophthalmic examination. Refraction tests, visual acuity measurement, central corneal thickness (CCT), Goldmann applanation tonometry, slit lamp examination, and dilated stereoscopic examination of the optic disc were performed in all subjects. Additionally, the retinal nerve fibre layer (RNFL) thickness was measured using the circular scan protocol of the SD-OCT (Spectralis, Heidelberg Engineering GmbH, Heidelberg, Germany) and the optic nerve head (ONH) was examined using Heidelberg Retina Tomography (HRT 3; Heidelberg Engineering GmbH, Heidelberg, Germany). Standard automated perimetry (Humphrey Field Analyser II 750; 24–2 Swedish interactive threshold algorithm; Carl Zeiss Meditec, Inc., Dublin, CA) was performed in all subjects. A reliable visual field test was defined as one with less than 25% fixation loss and less than 30% false positives and negatives. Subjects were excluded if they had a history of ocular surgery within 12 months before the onset of the study. Patients younger than 40 years old, with intraocular disease (e.g., macular degeneration, diabetic retinopathy, retinal vein occlusion) or neurological disorders affecting visual fields were also excluded from the study. We also excluded eyes with spherical equivalent of < − 6.0 D or > + 3.0 D, and cylinder correction of < − 3.0 D or > + 3.0 D. In the case when both eyes met the inclusion criteria the eye for the study was chosen randomly. Finally, patients with hypotension, severe circulatory failure or other vascular endothelial abnormalities, which could impact the optic nerve head blood flow, were also excluded from the study.

All patients were Caucasian. Patients were assigned to three groups:

1. **Healthy Controls (HC)**–subjects having uncorrected IOP of ≤ 22 mmHg, healthy optic disc appearance and normal visual field. A normal visual field was defined as the absence of glaucomatous and neurologic field defects.
2. **Normotensive Glaucoma Suspects (NGS)**–subjects having uncorrected IOP of ≤ 21 mmHg with eyes having glaucomatous optic disc appearance (GODA), but normal visual fields. GODA was defined as i) disc rim loss or excavation, ii) optic disc cupping (C/D > 0.6 assessed subjectively) or iii) localized abnormalities of the RNFL.
3. **Primary Open Angle Glaucoma (POAG)**–subjects having clearly defined presence of glaucomatous optic nerve damage (i.e., concentric enlargement of the optic disc, rim thinning, or notching) with associated visual field defects and high or normal IOP in the presence of an open angle.

Patients were taking beta-blocker drops (10% and 60%, in NGS and POAG group, respectively), prostaglandins (25% and 61%, in NGS and POAG group, respectively), carbonic anhydrase inhibitor eye drops (1% and 33%, in NGS and POAG group, respectively), and alpha antagonists (3% and 21%, in NGS and POAG group, respectively). All the values were rounded to nearest percent.

We carefully reviewed the information from the medical history of patients with hypertension and reported that in the group of HC 21 out of 69 subjects were taking oral medications: beta-blockers (38%), Ca channel antagonists (28%) angiotensin converting enzyme inhibitors (28%) and angiotensin receptor antagonists (19%). In the NGS group, 18 out of 72 patients were taking oral medications: beta-blockers (50%), Ca channel antagonists (22%) angiotensin converting enzyme inhibitors (22%) and angiotensin receptor antagonists (11%). In the POAG group, 14 out of 70 patients were taking oral medications: beta-blockers (64%), Ca channel antagonists (28%) angiotensin converting enzyme inhibitors (14%) and angiotensin receptor antagonists (14%). Again all the values were rounded to the nearest percent.

### Colour Doppler imaging measurements

**Fig 1:**
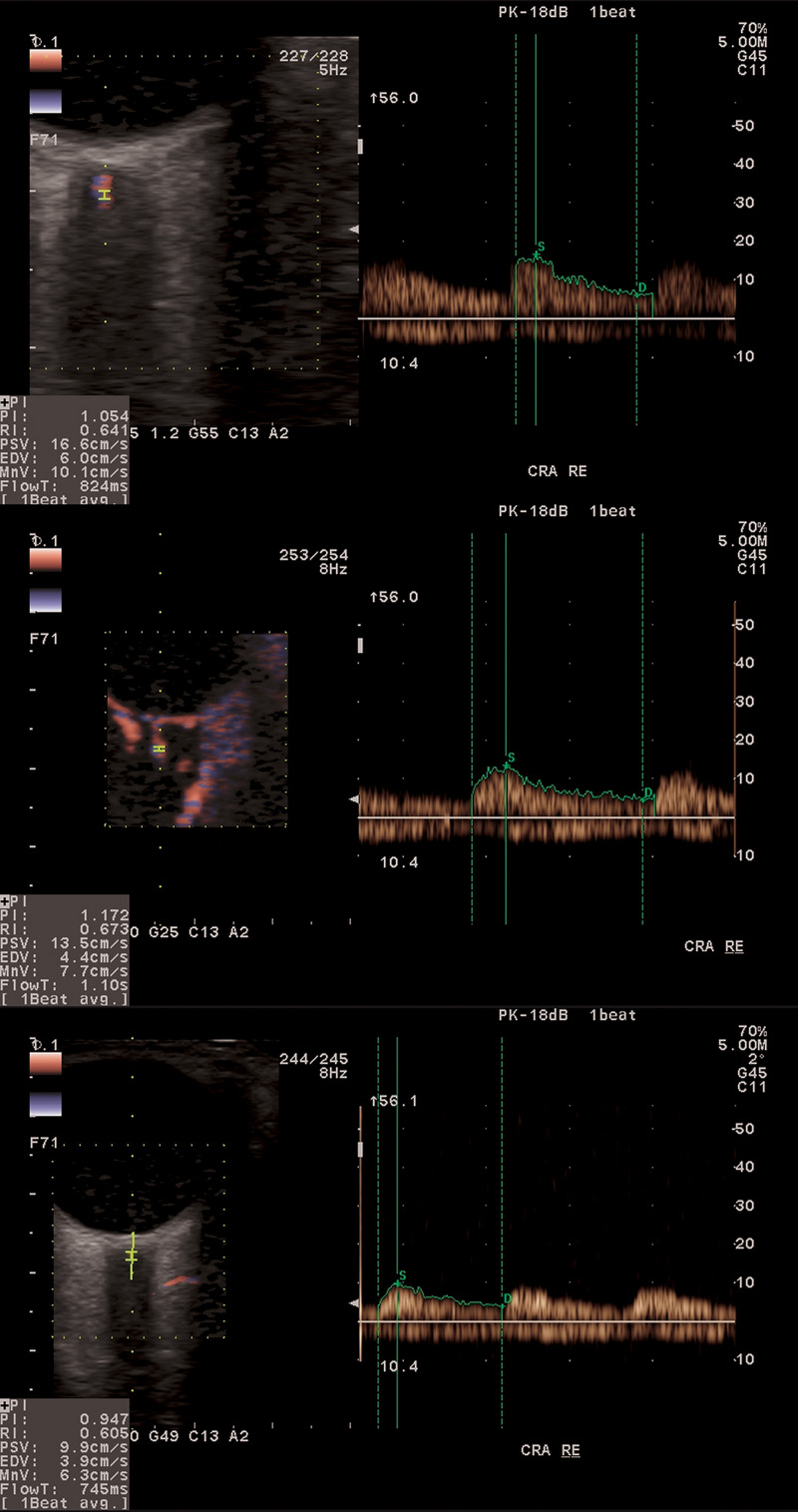
An example of ocular blood flow in CRA for the three considered groups of subjects. From top to bottom: healthy control, normotensive glaucoma suspect, glaucoma patient.

Aloka ProSound Alpha 6 (Hitachi Prosound Alpha 6; Aloka Co., Ltd., Japan) equipped with an eFlow–a high-definition ocular blood flow (OBF) imaging mode–was used to obtain CDI measurements of the ophthalmic artery (OA), central retinal artery (CRA), short posterior ciliary arteries (nasal and temporal, NPCA and TPCA, respectively) in each subject. Fig 1 shows three typical examples of OBF measurement in CRA for control subject (top), glaucoma suspect (middle), and glaucoma patient (bottom). The eFlow function displays blood flow information with higher sensitivity and resolution than with conventional methods and provides detailed visualization of the smallest vessels. With the patient in supine position, after a few minutes of rest, sterile ophthalmic gel was applied at the closed eyelid, and the 8.0 MHz linear probe was positioned gently with minimal pressure. Special care was taken for the OBF measurements to be of high quality. All OBF measurements were performed by the same clinician (K.C.), masked in terms of group allocation, who strived to accurately and consistently locate the probe ensuring the correct angle position in all the measurements. A constant test protocol was retained during the study avoiding unintended pressure of the probe on the eyeball during the test and maintaining constant distance of sampling gate in the time of consecutive measurements. NPCA and TPCA were measured at a position that was close to the ONH and as anterior as possible from the choroid not to receive any interference. A pilot study, conducted on several healthy subjects, confirmed reproducibility of acquired results. Appropriate angle correction was used for the accuracy of the velocity measurements as described previously [19]. Peak systolic velocity (PSV), defined as the highest velocity of the blood flow during the systolic phase of the cardiac cycle and end diastolic velocity (EDV), defined as velocity of blood flow at the end of diastolic phase of the cardiac cycle were recorded for each blood vessel. Additionally, the mean flow velocity (V_m_), the pulsatility index (PI) and resistivity index (RI) were evaluated.

Digital automatic blood pressure (BP) monitor (Omron M6 HealthCare Co., Ltd., Japan) was used to provide systolic and diastolic pressure readings, as well as pulse rate. The BP measurements were obtained immediately before investigation of the retrobulbar blood flow. The mean arterial pressure (MAP) was calculated as 1/3 systolic BP + 2/3 diastolic BP and the mean ocular perfusion pressure (MOPP) was calculated using the formula: MOPP = 2/3 (MAP − IOP).

### Image acquisition protocol of LC

A set of horizontal B-scans of LC were obtained using Spectralis spectral domain (SD) OCT system. The OCT device was set to image a 15°×10° rectangle centred on the optic disc using the enhanced depth imaging technique. This rectangle was scanned with approximately 75 B-scans separated by 30 to 34 μm (the scan line distance was determined automatically by the instrument). Approximately 42 SD-OCT frames were averaged for each section. The SD-OCT images were acceptable for the study only when the quality score was higher than 18.

### Analysis of the LC parameters

The details of this measurement method have been described previously [17]. In brief, all image processing procedures have been custom written in Matlab (MathWorks, Inc., Natick, MA, USA). Two points characterizing the Bruch’s membrane opening (BMO) and eight points describing the anterior LC were manually marked for each OCT image from a set of 16 images in each measurement using a specially designed graphic user interface, hence 3376 scans in total were analysed. The manually delineated points of BMO and those of LC were used to estimate lamina cribrosa depth (LCD)–distance between the line joining the two points of BMO and the farthest (deepest) point of LC, lamina cribrosa deflection depth (LCDD)–distance between the line joining the two extreme points of LC and the farthest (deepest) point of LC, lamina cribrosa shape index (LCSI)–characterizing a three-dimensional curvature of LC and the lamina cribrosa shape index in a horizontal direction (LCSI_H_)–characterizing two-dimensional curvature of LC in the central horizontal direction.

### Numerical and statistical analysis

Mean values and corresponding standard deviations were used along the non-parametric ANOVA (Kruskal-Wallis test) as normality of most of the parameter samples were rejected (Shapiro-Wilk test, *P* > 0.05). Post-hoc analysis included the Mann-Whitney test with a Bonferroni correction. Univariate linear regression was used to determine factors associated with the OBF velocities and the LC parameters. Partial correlation analysis was used to account for Age, CCT and IOP. Statistical analyses were performed using Matlab (MathWorks, Natick, MA). Value of *P* < 0.05 were considered significant.

## Results

This study included 211 selected participants (141 females, 70 males) with an age range of 42 to 86 years from consecutive patients who presented at the time of the study at the Outpatient and Glaucoma Clinic at the Department of Ophthalmology, Wroclaw Medical University. Patients were divided into 3 groups: 69 healthy controls (HC), 72 normotensive glaucoma suspects (NGS) and 70 POAG patients. The results for the group analysis are presented in Table 1. All parameters presented in Table 1 including LC evaluation were measured at the time of vascular assessment.

**Table 1:** Group mean (± one standard deviation, SD) results for the considered parameters and the results of ANOVA test (*P* value).

Variables	HC (*n* = 69)	NGS (*n* = 72)	POAG (*n* = 70)	*P* value (ANOVA)
Mean age (years)	65.0 ± 9.0	67.6 ± 8.0	68.0 ± 8.0	0.078
Males (*n*)/Females (*n*)	23/46	19/53	28/42	-
Mean CCT (μm)	562 ± 39	547 ± 31	542 ± 33	**0.002**†
Mean IOP (mmHg)	17.0 ± 3.0	17.0 ± 3.0	16.0 ± 3.0	0.446
Mean VF MD (dB)	−0.37 ± 1.04	−0.28 ± 1.12	−8.64 ± 7.71	**<0.001**‡
Mean VF PSD (dB)	1.64 ± 0.42	1.60 ± 0.32	6.61 ± 3.95	**<0.001**‡
Disc area (HRT parameter) (mm^2^)	2.26 ± 0.40	2.31 ± 0.34	2.12 ± 0.32	**0.005**‡
Rim area (HRT parameter) (mm^2^)	1.61 ± 0.31	1.16 ± 0.21	0.87 ± 0.31	**<0.001**§
Rim volume (HRT parameter) (mm^3^)	0.43 ± 0.11	0.25 ± 0.09	0.16 ± 0.10	**<0.001**§
Average RNFL thickness (μm)	96.5 ± 8.3	86.4 ± 10.1	64.0 ± 12.6	**<0.001**§
Temporal Superior RNFL (μm)	135.2 ± 14.6	119.6 ± 20.5	80.6 ± 24.1	**<0.001**§
Temporal RNFL (μm)	70.2 ± 10.4	64.5 ± 10.8	50.6 ± 13.4	**<0.001**§
Temporal Inferior RNFL (μm)	141.8 ± 17.7	123.1 ± 22.7	75.3 ± 29.5	**<0.001**§
Nasal Superior RNFL (μm)	104.2 ± 20.5	90.2 ± 17.5	70.7 ± 18.0	**<0.001**§
Nasal RNFL (μm)	72.1 ± 11.6	66.0 ± 12.1	56.1 ± 16.1	**<0.001**§
Nasal Inferior RNFL (μm)	108.2 ± 19.7	98.0 ± 18.9	72.2 ± 22.4	**<0.001**§
LCD (μm)	532 ± 116	563 ± 117	600 ± 173	**0.015**¶
LCDD	224 ± 59	216 ± 56	241 ± 68	**0.048**¶¶
LCSI	0.36 ± 0.13	0.33 ± 0.17	0.35 ± 0.15	0.357
LCSI_H	−0.19 ± 0.36	−0.02 ± 0.42	−0.02 ± 0.41	**0.015**†
Systolic BP (mmHg)	143 ± 17	145 ± 17	145 ± 20	0.744
Diastolic BP (mmHg)	86 ± 10	86 ± 10	89 ± 12	0.113
MAP (mmHg)	105 ± 11	106 ± 11	108 ± 14	0.383
MOPP (mmHg)	59 ± 8	59 ± 7	61 ± 9	0.229
Hypertension (*n*)	21	18	14	-

HC = healthy controls; NGS = normotensive glaucoma suspects; POAG = primary open-angle glaucoma; CCT = central corneal thickness; IOP = intraocular pressure; VF MD = visual field mean deviation; VF PSD = visual field pattern standard deviation; HRT = Heidelberg Retina Tomography; RNFL = retinal nerve fiber layer; LCD = lamina cribrosa depth; LCDD = lamina cribrosa deflection depth; LCSI = lamina cribrosa shape index; LCSI_ H = lamina cribrosa shape index in a horizontal direction; BP = blood pressure; MAP = mean arterial pressure; MOPP = mean ocular perfusion pressure; values with statistical significance are shown in bold

† P value is significant between normal and suspects and between normal and POAG.

‡ P value is significant between normal and POAG and between suspects and POAG.

§ P value is significant among all groups.

¶ P value is significant between normal and POAG.

¶¶ P value is significant between suspects and POAG.

All P values including with Bonferroni correction (α<0.05/3).

The results of ONH and RNFL parameters for NGS fall, on average, in between those of healthy controls and POAG patients. In this study, the rim area and rim volume differentiated well normotensive glaucoma suspects from POAG patients and healthy controls (ANOVA, both *P* < 0.001). Also, NGS had significantly thinner RNFL thickness from healthy subjects in average and in all six sectors (ANOVA, *P* < 0.001).

Concerning ocular blood flow biomarkers, results from the CDI variables are presented in Table 2.

**Table 2:** Ocular blood flow biomarkers in orbital vessels obtained using color Doppler Imaging device (mean ± one standard deviation, SD) and the results of ANOVA test (*P* value).

Variables	HC (*n* = 69)	NGS (*n* = 72)	POAG (*n* = 70)	*P* value (ANOVA)
OA, PSV, cm/s	35.59 ± 8.9	34.18 ± 8.9	30.66 ± 8.9	**0.004**†
OA, EDV, cm/s	8.26 ± 3.2	8.30 ± 3.5	7.48 ± 2.9	0.233
OA, V_m_, cm/s	17.81 ± 5.5	16.95 ± 5.5	15.38 ± 4.9	**0.026**†
OA, PI	1.57 ± 0.3	1.57 ± 0.3	1.55 ± 0.4	0.889
OA, RI	0.77 ± 0.1	0.76 ± 0.1	0.75 ± 0.1	0.401
CRA, PSV, cm/s	14.28 ± 2.8	13.85 ± 3.2	12.55 ± 2.9	**0.002**‡
CRA, EDV, cm/s	4.04 ± 1.0	4.16 ± 0.9	3.92 ± 1.2	0.404
CRA, V_m_, cm/s	7.84 ± 1.5	7.74 ± 1.6	7.12 ± 1.6	**0.017‡**
CRA, PI	1.30 ± 0.3	1.26 ± 0.3	1.22 ± 0.3	0.175
CRA, RI	0.71 ± 0.1	0.69 ± 0.1	0.68 ± 0.1	0.112
NPCA, PSV, cm/s	12.99 ± 2.6	13.74 ± 3.1	13.19 ± 3.6	0.339
NPCA, EDV, cm/s	4.09 ± 1.4	4.47 ± 1.4	4.20 ± 1.1	0.215
NPCA, V_m_, cm/s	7.80 ± 1.5	8.20 ± 1.7	7.73 ± 1.8	0.204
NPCA, PI	1.16 ± 0.3	1.14 ± 0.2	1.15 ± 0.2	0.938
NPCA, RI	0.68 ± 0.1	0.67 ± 0.1	0.67 ± 0.1	0.860
TPCA, PSV, cm/s	14.30 ± 2.6	13.20 ± 2.9	13.14 ± 3.2	0.050
TPCA, EDV, cm/s	4.31 ± 1.4	4.07 ± 1.2	4.23 ± 1.4	0.558
TPCA, V_m_, cm/s	8.34 ± 1.7	7.77 ± 1.5	7.81 ± 1.6	0.063
TPCA, PI	1.20 ± 0.3	1.18 ± 0.3	1.14 ± 0.3	0.511
TPCA, RI	0.69 ± 0.1	0.68 ± 0.1	0.67 ± 0.1	0.551

HC = healthy controls; NGS = normotensive glaucoma suspects; POAG = primary open-angle glaucoma; OA = ophthalmic artery; CRA = central retinal artery; NPCA = nasal short posterior ciliary artery; TPCA = temporal short posterior ciliary artery; PSV = peak systolic velocity; EDV = end diastolic velocity; V_m_ = mean flow velocity; PI = pulsatility index; RI = resistance index; values with statistical significance are shown in bold

† P value is significant between normal and POAG.

**‡**P value is significant between normal and POAG and between suspects and POAG.

All P values including with Bonferroni correction (α<0.05/3).

As expected, ocular blood flow biomarkers in POAG patients were statistically significantly reduced when compared to healthy controls. In post-hoc analysis, this was observed in PSV (*P* = 0.001 in OA and *P* < 0.001 in CRA) and V_m_ (*P* = 0.008 in OA and *P* = 0.008 in CRA). OBF biomarkers in POAG, in terms of PSV and V_m_, were not statistically significantly different to that of NGS except for PSV in CRA (*P* = 0.011). In TPCA, when compared to HC, reduced values of PSV and V_m_ were obtained for the NGS and POAG groups, but these results were on the verge of statistical significance (*P* = 0.05 and *P* = 0.063, respectively). In NGS patients the mean OBF biomarkers were also reduced in the majority of parameters, but these values fell, on average, between those of healthy controls and glaucoma patients.

**Fig 2:**
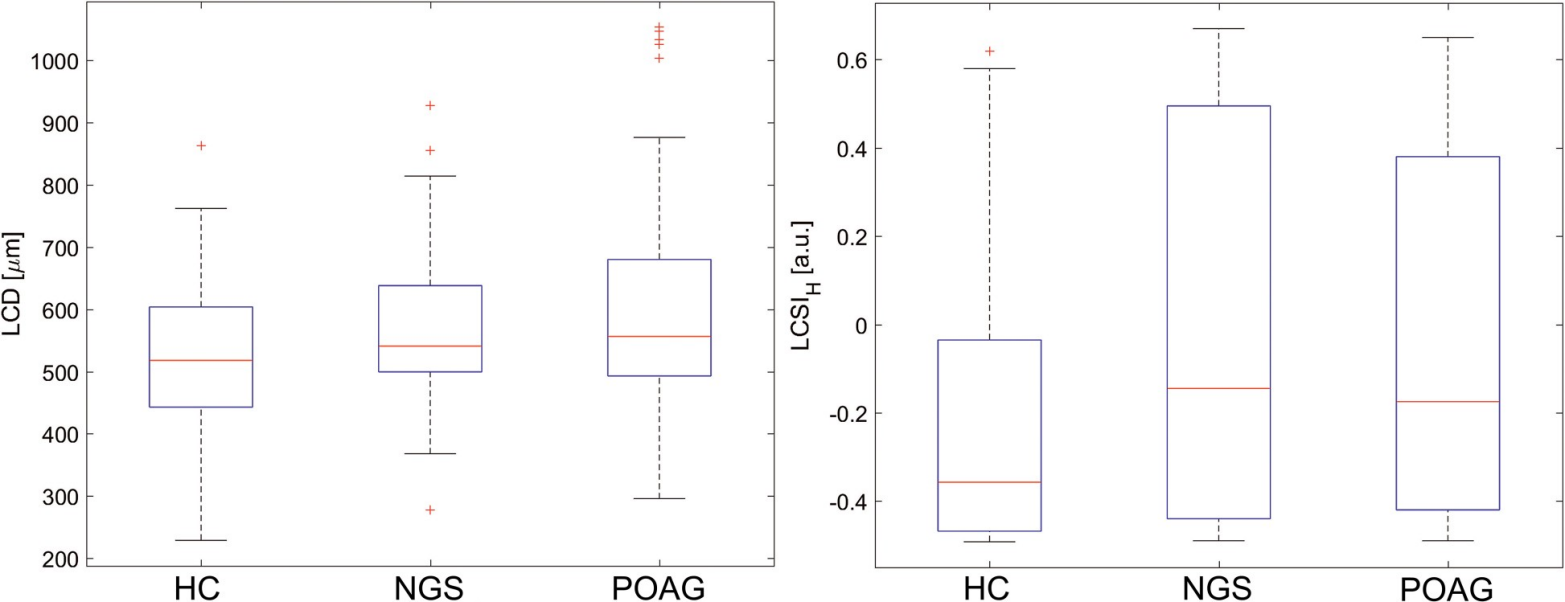
The boxplots for LCD (left) and LCSI_H_ (right) for the three considered groups of subjects. Red crosses denote outliers.

Regarding the LC parameters comparison between the groups, statistically significantly posterior displacement of the anterior surface LC (LCD) was found in POAG patients as compared to healthy controls (*P* = 0.004). The LCD group average of normotensive glaucoma suspects was found to be situated in between the LCD group averages of POAG patients and healthy controls. Measurement of LC Deflection Depth parameter (LCDD) varied POAG patients from the NGS subjects (*P* = 0.0016), but failed to differentiate between healthy controls and NGS group (*P* = 0.333). LCSI was statistically insignificant between the groups (*P* = 0.357). However, LCSI_H_ parameter showed that POAG patients and NGS subjects have a similar shape of LC in the central part of the anterior surface (*P* = 0.333), but statistically significantly different from that of healthy controls (*P* = 0.014 and *P* = 0.010, respectively). Fig 2 shows the boxplots for the LCD and LCSI_H_.

A statistically significant correlation corrected for age, CCT, IOP and the presence of hypertension was found in POAG patients between several LC parameters and OBF biomarkers (see Table 3). No significant correlation was observed between these parameters in NGS subjects and healthy controls.

**Table 3:** Correlations corrected for age, central corneal thickness, intraocular pressure and the presence of hypertension between LC parameters and OBF biomarkers.

Group	LC parameter	OBF biomarker	Vessel	*R*	*P* value
POAG	LCSI_H_	EDV	CRA	0.315	**0.010**
POAG	LCSI_H_	V_m_	CRA	0.319	**0.009**
POAG	LCSI_H_	EDV	NPCA	0.285	**0.020**
POAG	LCSI_H_	V_m_	NPCA	0.346	**0.005**
POAG	LCSI	PSV	NPCA	−0.333	**0.006**
POAG	LCSI	V_m_	NPCA	−0.294	**0.016**
POAG	LCSI	PI	NPCA	−0.270	**0.029**

LC = lamina cribrosa; OBF = ocular blood flow; POAG = primary open-angle glaucoma; LCSI_ H = lamina cribrosa shape index in a horizontal direction; LCSI = lamina cribrosa shape index; CRA = central retinal artery; NPCA = nasal short posterior ciliary artery; PSV = peak systolic velocity; EDV = end diastolic velocity; V_m_ = mean flow velocity; PI = pulsatility index; RI = resistance index; values with statistical significance (ANOVA) are shown in bold.

Statistically significant correlations, corrected for age, CCT, IOP and the presence of hypertension, were found in POAG patients between nasal-superior RNFL thickness and OBF biomarkers (see Table 4). No significant correlation was observed between these parameters in NGS subjects and healthy controls.

**Table 4:** Correlations corrected for age, central corneal thickness, intraocular pressure and the presence of hypertension between RNFL thickness and OBF biomarkers.

Group	RNFL thickness	OBF biomarker	Vessel	*R*	*P* value
POAG	NS	PSV	CRA	0.306	**0.013**
POAG	NS	EDV	CRA	0.296	**0.016**
POAG	NS	V_m_	CRA	0.277	**0.024**
POAG	NS	PSV	NPCA	0.271	**0.028**
POAG	NS	EDV	NPCA	0.450	**<0.001**
POAG	NS	V_m_	NPCA	0.357	**0.003**
POAG	NS	PSV	TPCA	0.301	**0.014**
POAG	NS	EDV	TPCA	0.293	**0.017**
POAG	NS	V_m_	TPCA	0.325	**0.008**

RNFL = retinal nerve fiber layer; OBF = ocular blood flow; POAG = primary open-angle glaucoma; NS = nasal superior; CRA = central retinal artery; NPCA = nasal short posterior ciliary artery; TPCA = temporal short posterior ciliary artery; PSV = peak systolic velocity; EDV = end diastolic velocity; V_m_ = mean flow velocity; values with statistical significance (ANOVA) are shown in bold

Additionally, for NGS subjects statistically significant correlations were found between MAP (and MOPP) and PSV, EDV, and V_m_ of NPCA (*R* = 0.347 with *P* = 0.003, *R* = 0.240 with *P* = 0.047, *R* = 0.343 with *P* = 0.004 respectively). No such correlations were found for the POAG group whereas for the healthy controls this was evident only for EDV in both NPCA (*R* = 0.260; *P* = 0.035) and TPCA (*R* = 0.275; *P* = 0.025).

## Discussion

Patients suspected of having glaucoma based on glaucomatous optic disc appearance represent a group of individuals who pose a diagnostic dilemma. Recently, we demonstrated that in glaucoma suspects early changes in the shape of LC can be observed, mainly in its central part, despite the low (nominal) intraocular pressure [17]. These changes had a similar character to those observed in POAG patients.

In this study, focus was made on OBF biomarkers in normotensive glaucoma suspects and for the first time, on the relationship between these biomarkers and LC features in this particular group. Previous studies determined the OBF biomarkers primarily in comparison between healthy and glaucomatous eyes [20–23] or considered the impact of age on retrobulbar flow [24–26]. Similarly to other studies [8, 20, 21] we confirmed the statistically significantly reduced mean PSV biomarker in the OA and CRA in POAG patients. Meta-analysis of CDI parameters performed by Meng et al. [22] showed, that significant reduction of this variable in the OA and CRA was the most frequently found result in the group of patients with POAG. This variable was also found to be the most relevant to clinical outcomes such as diagnosis and progression of glaucoma. Also, in our study V_m_ of OA and CRA was found to be statistically significantly decreased in POAG patients as compared to healthy controls and this is in an agreement with study of Garhöfer et al. [21] While the IOP was not different between the groups at the time of the study, the reduction in the OBF biomarkers of POAG patients may be in response to elevated IOP before appropriate treatment was started, influenced by the impaired autoregulatory system, which determines an individual susceptibility to optic nerve damage.

Although reduced values of OBF biomarkers in POAG patients have been confirmed in many studies, publications on glaucoma suspects are less well established. Calvo et al. followed glaucoma suspects with GODA for 48-month period and stated that participants who converted to glaucoma had significantly lower EDV and V_m_ in OA [9]. However, the group of suspects they studied had increased IOP. Interestingly, Calvo et al. [9] did not demonstrate abnormalities in the OBF biomarkers in CRA or short posterior ciliary arteries and explained their results with the higher variability of OBF biomarkers when compared to the OA. In our study, normotensive glaucoma suspects had lower PSV and V_m_ in OA and CRA. However, they did not reach the statistical significance when compared to other study groups. Also, they had IOP within normal limits and MOPP results close to healthy controls. It would be interesting to find whether fluctuations in IOP in glaucoma suspected eyes affect the flow reduction in ONH. Deokule et al. [8] on a small study group, did not observe a decrease in the OA, CRA and TPCA parameters in glaucoma suspects compared to glaucoma patients and healthy controls using CDI. They stressed that they were unable to obtain good quality images of NPCA due to the smaller size of the vessel and greater anatomic variability. In our study, we obtained high quality repeatable images of NPCA, confirmed in a pilot study on healthy subjects, likely because of the eFlow function of CDI, which provides detailed visualization of the smallest vessels. However, we were unable to show statistically significant differences between the groups in NPCA and TPCA.

As the lamina cribrosa region is nourished by branches arising directly from the short posterior ciliary arteries, which penetrate through the peripapillary sclera to form a capillary plexus in LC [27], and physiological obstruction around the LC has been suggested to be hemodynamically significant [28], we decided to evaluate whether deformation of lamina cribrosa detected *in vivo* by OCT is related to the blood supply in this region.

To the best of our knowledge, this is the first study to evaluate the relationship between OBF biomarkers and LC parameters in normotensive glaucoma suspects as compared to glaucoma patients and healthy controls. Our study revealed significant correlations between the posterior deformation of the central LC and EDV and V_m_ of CRA and NPCA in POAG patients. Also, all examined parameters of NPCA except EDV presented significant correlations with the mean shape of LC. These results confirm a close relationship between the LC shape undergoing glaucoma deformation and the flow reduction in the short posterior ciliary artery supplying the anterior ONH. Our outcomes were corrected for age according to the work of Embleton et al. [18], who reported that capillary and lamina cribrosa blood volume decreases with age.

Although LCD and LCSI_H_ parameters in NGS subjects had similar appearance to that of glaucoma patients and it was markedly different from that of healthy controls, we were unable to show correlation between the LC and OBF parameters. It is widely accepted that the short posterior ciliary arteries represent the main source of blood for the anterior optic nerve [27]. Existing evidence suggests effective autoregulation of blood flow to the anterior ONH to a significant extent in IOP [29]. Possibly the lack of correlation between LC glaucomatous shape and OBF biomarkers is related to low IOP in these eyes and partially the autoregulation system, which allow these patients to maintain relatively constant flow despite fluctuations in the perfusion pressure [30–32]. Previous study demonstrated evidence for impairment of such regulatory function in short posterior ciliary arteries in response to acute, large elevations in IOP [33]. Our glaucoma suspects were all normotensive patients with glaucomatous optic disc appearance and normal visual field.

This study has some limitations. Only one vascular bed was analysed in this study, hence information on choroidal or retinal blood flow that could contribute to the relationships between the LC parameters and OBF biomarkers was not available. Also, although IOP was taken in the correlation analysis as a control variable, no information was available for the glaucoma group on the original levels of IOP that influenced both the LC shape and position as well as the OBF biomarkers because all patients were on antiglaucoma medications (no washout). Hence, studies with untreated glaucoma patients are of interest [34]. The spectral OCT used in this study could not successfully image the posterior surface of lamina cribrosa, prohibiting the measurement of its thickness, which could shed more light into potential compression of the ciliary vessels within the lamina cribrosa. The use of a swept-source OCT could alleviate this problem. Another potential limitation is that a small percentage of patients could have had nocturnal pressure drops. Since 24h monitoring of blood pressure was not exercised, the potential nocturnal hypotension could not be clearly excluded.

In conclusion, this study provides the evidence for the relationship between LC shape and reduction of OBF biomarkers in glaucoma patients. Although our initial hypothesis that the association of OBF biomarkers with morphological features in the region of ONH in the group of NGS is similar to that of POAG patients was rejected, our results support the postulate that impaired blood flow is observed with LC deformation in glaucoma patients and may play an important role in a disease progression. Although mean shape of the LC in normotensive glaucoma suspects had similar appearance to that of glaucoma patients we did not show its correlation with OBF biomarkers.

## Supporting information

S1 File (XLSX)
